# SciBabel: a system for crowd-sourced validation of automatic translations of scientific texts

**DOI:** 10.5808/GI.2020.18.2.e21

**Published:** 2020-06-15

**Authors:** Felipe Soares, Rozane Rebechi, Mark Stevenson

**Affiliations:** 1 Computer Science Department, University of Sheffield, Sheffield S38RA, UK; 2 Instituto de Letras, Universidade Federal do Rio Grande do Sul, Porto Alegre 91540-000, Brazil

**Keywords:** crowdsourcing, linguistics, machine translation, medical informatics applications, PubMed

## Abstract

Scientific research is mostly published in English, regardless of the researcher's nationality. However, this growing practice impairs or hinders the comprehension of professionals who depend on the results of these studies to provide adequate care for their patients. We suggest that machine translation (MT) can be used as a way of providing useful translation for biomedical articles, even though the translation itself may not be fluent. To tackle possible mistranslation that can harm a patient, we resort to crowd-sourced validation of translations. We developed a prototype of MT validation and edition, where users can vote for that translation as valid, or suggest modifications (i.e., post-editing the MT). A glossary match system is also included, aiming at terminology consistency.

**Availability:** Available online under the MIT license at https://github.com/soares-f/scibabel.

## Introduction

Research in the biomedical domain, particularly about treatments and procedures for humans, can help improve the patient care offered by physicians. Evidence-based medicine is based on the premise that physicians give the best care possible when they base their treatments on reliable scientific evidence. But, although in practice this access is possible, there is a limitation that makes evidence-based medicine out of the reach for many physicians: almost all of its contents are written in English.

During the first half of the 20th century, scientific research was published in a variety of languages. But, as Gordin [1] described in detail, a complex set of factors led to English becoming the language of most scientific publications following the Second World War. Researchers tend to publish in English regardless of their native language. But, while academic researchers are often proficient in English, this may not be true for physicians in non-English speaking countries.

Translation of documents into the languages with which physicians are familiar seems like an obvious way to make the world’s scientific production accessible to them. But new research is produced so quickly and its results are published so rapidly that translating the information manually would be impractical. For example, in 2019 alone, more than 10,000 new articles were published in PubMed (PubMed Query: ((("2019"[Date - Publication] : "3000"[Date - Publication])) AND (treatment[Title/Abstract])) AND (procedure[Title/Abstract])) containing the keywords “treatment” and “procedure”—exactly the kind of articles that would be of interest to physicians. However, there is a technology that could potentially do this translation automatically: machine translation (MT).

MT is a technology to render texts written in one language to another language. Modern MT research began just after the Second World War with the automatic translation of Russian scientific texts to English [2] as part of the scientific response to the Cold War (e.g., see Hutchins [3]). Machine translation research fell into decline soon thereafter due to considerable skepticism about whether practical MT systems were possible within the research community [4], but MT resurged in the 1990s with the advent of more powerful computers and alternative approaches. The field of MT experienced explosive growth after the September 2001 terrorist attacks and is an active area of scientific research [5-8]. This effort has led to a substantial improvement in the quality of translations produced by MT systems [9].

The earliest work on MT for scientific content concentrated on the physical sciences, however the focus of current research is shifting towards biomedical texts, especially due to shared tasks. This difference is important because, while users of translations in other scientific fields can tolerate some amount of error, as they do not have such a strict vocabulary and are not dealing directly with human beings, even a small mistranslation in this domain (e.g., a drug name being incorrectly translated, or a negation being ignored) could lead to disastrous consequences to patients. For example, consider Supplementary Table 1 which shows examples of a simple medical instruction (i.e., “Take two pills orally every day unless you feel dizzy or lightheaded”) usually found in drugs prescriptions translated into Finnish, Korean, Portuguese, Italian, Spanish, Japanese, French, German, Russian, Chinese (simplified) and Ukrainian by Google Translate. The third column contains their translations back into English by an educated native speaker (a common method of evaluating MT, similar to an approach known as back-translation) [10]. Contraindications that have been incorrectly translated are highlighted in bold font and it can be seen that these occur in six of the 11 translations. This demonstrates the need for automatic translations to be manually checked for critical mistranslations. However, this process is time-consuming and unlikely to scale well. Therefore, we propose a crowd-sourced approach to validate automatic translations of biomedical articles and develop a prototype to facilitate such task.

In the proposed system volunteers who are able to read biomedical articles in English and also in another language would check MT output for critical mistranslations and vocabulary adequacy. The purpose of this system is to guarantee that the message in the source text is correctly conveyed in the translation, even though the translated text may lack fluency. Volunteers would accept the proposed translations if they are correct and be able to make editions when appropriate (e.g., incorrect terminology). We expect that our system, named SciBabel, would allow physicians and medical staff not proficient in English to access the most recent advances in medicine, enabling them to provide their patients with better treatment. The source code is available at https://github.com/soares-f/scibabel.

## Background

An illustration of the recent improvements in MT can be seen from the performance of systems reported in the biomedical track of the Conference on Machine Translation (WMT), which focuses on the translation of PubMed abstracts. Translation quality increased by around 51% (or 16 percentage points) from 2016 to 2019 for English to Spanish. In Table 1, we show the MT performance for some language pairs for biomedical texts with dates ranging from 2013 to 2019 for selected language pairs. Note that translation quality is measured automatically using the BLEU score, a common MT metric that relies on the overlapping portions of the generated translations and the manually translated text [17].

In the two most recent WMT conferences (2018 and 2019) interesting results were reported for the English/Portuguese and English/Spanish language pairs. For instance, for the English to Spanish, the number of MT-generated sentences judged by humans as better than human translations was larger than the number of human sentences judged better than MT ones. When combining the number of times that the best MT was equally good or better than human translation for WMT19, we get an average of 73% of correct translations according to human judgment, with surprising 90% for EN/ES and 82.09% for ZH/EN. This strengthens our point that MT can indeed be used to aid dissemination of biomedical scientific content.

However, as shown in Supplementary Table 1, MT systems can make critical mistakes when considering the usage of a medicine, for instance. It has been shown in literature that even human translation is prone to errors [18]. That is why the translation and localization industry usually has a two-step (or even more) process for translation. That is, at least one additional human is involved in checking the translation already carried out (also called proof-reading) [19].

Crowdsourcing of intensive tasks is not new in science. One example can be the Folding@Home initiative [20], which was popular in the first decade of the years 2000’. This initiative consisted of crowdsourcing computational power from regular end-users (that signed to the initiative) to simulate protein folding, drug design, and molecular dynamics. Similarly, Seti@Home [21] tried to follow the same path to search for extraterrestrial life.

The crowdsourcing of manual annotation (or evaluation) was already explored by different authors [22,23]. For instance, the information retrieval (IR) shared tasks can be seen as the pioneers of human distributed annotation. Participants of IR shared tasks would blindly evaluate the participants’ automatic predictions. Another example of distributed annotation is the Amazon Mechanical Turk, which pays users to manually annotate tasks. Some authors developed games [24-26] or mobile apps [27] to gather human annotation.

Regarding crowdsourcing of translations, Zaidan and Callison-Burch [28] state that collecting translations by crowdsourcing using non-professionals may lead to low-quality results. They propose the use of distance among translations and LM perplexity to score collected translations to discriminate between “good” and “bad” translations.

Ambati et al. [29] explored the challenges involved in crowdsourcing translation based on their experiments with Amazon Mechanical Turk. Their main findings regarding challenges are related to the large label space, that is, even though there is a finite number of possible translations for a single translations, there is a much larger space of acceptable sentences in the target space, but that may not be adequate or not style compliant. The second one is the small number of bilingual speakers for low-resourced languages. The third one is low quality, as most of the crowd-sourced translators are not professional linguists. Given this scenario, they proposed a framework based on phases to enhance the final quality of crowd-sourced translations. The first step of the translation is done by weak bilingual translators, translations which are revised by bilingual translators and the final step is done by monolinguals of the target language or bilinguals whose mother tongue is the target one. Considering the potential of crowd-sourced annotation, we aimed at developing a prototype of a system to enable the manual evaluation of automatic translations tailored to biomedical texts and post-edition. Our goal was to produce a simple yet usable interface to annotate translations as valid in the target language, while enabling users to make adjustments in the translation to correct possible mistakes.

## Design

When idealizing such a tool, we envisioned not to provide perfect and fluent translations, since that would require a considerable effort from users. We are rather interested in finding gross and dangerous MT mistakes, the ones that could completely hinder the interpretation of the article. That is, we are interested in assuring that the translated text conveys the same original message, even though it may not sound completely fluent for a native speaker.

We can see as an example the sentence “*Nehmen Sie jeden Tag zwei Tabletten ein, es sei denn, Ihnen ist schwindelig oder benommen*” in German. The direct translation, as seen in Supplementary Table 1, is “Take two pills every day by mouth unless you feel dizzy or lightheaded.” This may not sound natural, but it conveys the message that the dosage is two pills with a daily frequency and the contra-indication is if the person feels dizzy or lightheaded.

### Functionalities

The following functionalities were implemented:

‒ Parallel visualization of the original text and the machine translated version.

‒ A “voting” system that allows users to flag a particular translation as correct (similar to a “like” in social media).

‒ An option to edit a suggested translation, allowing users to correct possible mistranslations.

‒ Only the last translation is available, since this is deemed to be the one with best quality.

‒ When editing a translation, a terminology lookup is available. That is, for each matched string in the source text, the suggested translation is shown.

### Technical details

In our prototype we aim at providing a simple and easily upgradable interface for document validation and modification. The prototype is coded in Python 3 using the Flask microframework. Our choice of Flask is due to its simplicity regarding back-end and frontend, while being able to scale if required.

For the interface, we opted for the Bootstrap library (https://getbootstrap.com/), since it provides responsive mobile-ready frontend components. The functionalities were expanded using JQuery and Javascript.

As for the backend, we took advantage of the SQLAlchemy toolkit (https://www.sqlalchemy.org/), which is an ORM (Object Relational Mapper) that abstracts database operations. By using SQLAlchemy, we were able to make the app database agnostic. That is, the user can easily switch among the RDBMS supported by the package without needing to change several parts in the code.

Regarding the translation system behind the prototype, we used an in-house model developed with OpenNMT (https://opennmt.net/) which is decoupled from the interface. We do not think that at this point it is extremely relevant to have an online translation system, since new articles can be batch translated overnight, for instance.

For the dictionary, we encourage the usage of UMLS, since it is a very comprehensive asset, already standardized and is available in many languages. Users can also make use of SNOMED CT available in more than one language, when compatible with licensing.

## Results

We implemented our prototype following the design specified in Section 3. For such, we first created a simple interface to visualize the translated content in the source language (e.g., English in this case) and target language (e.g., French). In this first screen, bilingual users can check the translation, which is shown in column format. We also introduced a feature that allows users to hover over the source or target sentence and check which sentence it refers to on the other column of the parallel text. After checking the translation, bilingual users can flag (i.e., Like) the translation as good, or perform modifications (editing).

**Fig. 1.**
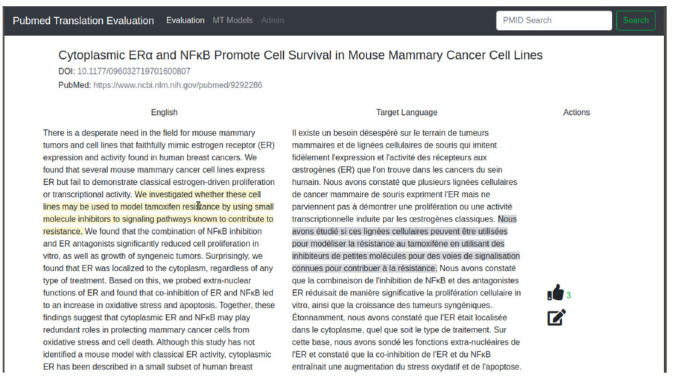
Interface for translation evaluation. Users can flag the translation as adequate (i.e., Like) or edit the proposed translation using the links in the Actions column.

In Fig. 1, we show a screenshot of the article validation step. We have already included placeholders in the top bar to allow inclusion of alternative MT models as well as access to an Administrator backend which is under development.

**Fig. 2.**
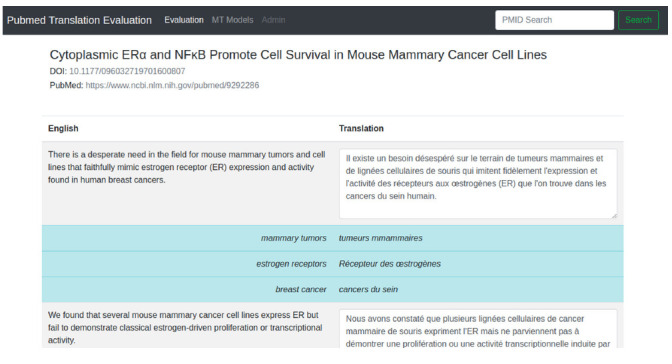
Interface for translation editions Users can edit the proposed translation to make corrections on mistranslations or terminology adequacy. The prototype also shows suggested translations from terms matched in a dictionary, aiming at providing terminology consistency.

In Fig. 2, we included a screenshot of the edition mode for the translated contents. In this view, the text is shown by sentences, with translations displayed as text boxes, such that users can perform post-edition on the suggested text. In addition, we included a glossary functionality, which can help users to guarantee terminology consistency. For this, a dictionary has to be supplied beforehand, and then a simple string matching is used to show the suggested translation. For instance, for the term “estrogen receptors”, the suggested translation in French is “Récepteur des œstrogènes”, while the automatic translation is “récepteurs aux œstrogènes”. Although the automatic translation is not wrong, the suggested term “Récepteur des œstrogènes” is flagged in UMLS (https://www.nlm.nih.gov/research/umls/index.html, Unified Medical Language System) as preferred.

## Conclusion and Further Steps

In this article, we pointed out the importance of making biomedical literature accessible to all healthcare professionals, despite the language they speak. As scientific publication, especially in biomedical sciences, has been fastly growing, manual translation of articles is an untractable approach to make such information multilingual. Thus, we argue that MT can be an alternative to alleviate such bottleneck.

However, despite the increasing performance of MT systems, some critical errors may occur when texts are translated, which can ultimately hinder patient safety. Thus, manual validation/evaluation of translations should be performed to mitigate potential risks. To enable validation to scale to several languages, we point out that crowdsourcing the effort may be a solution. Therefore, we developed a prototype of a system that can allow an easy translation validation and possible edition.

The prototype was developed using Python 3 and Flask (https://flask.palletsprojects.com/en/1.1.x/), with Bootstrap for the visual interface. A visualization and edition interface was created, and an Administrator interface is currently under development. We included visual features to help users when doing the validation or editing the text.

As future steps, we envision some important upgrades:

‒ Ability to export translations into TMX and TXML formats, since they are standard in the localization industry;

‒ Ability to flag different unit of measurements in translation (e.g., pounds to kilograms), since the numbers need to be converted accordingly;

‒ Include a voting scheme for rollback of manual edits and a “annotation” weight according to the mother tongue of the annotator. In addition, a similar approach for quality assurance as proposed by [29] could be used, by establishing a score for annotators as well as for annotations;

‒ Develop an additional view to allow annotation transfer between source and target languages.

The last upgrade, related to annotation transfer, can be extremely helpful to create multilingual annotated datasets by leveraging existing annotations in one language. For instance, one could use annotations already made in a document in English to transfer those annotations to a translated text, making annotation quicker and less expensive.

## Figures and Tables

**Table 1.** Machine translation performance in biomedical article abstract translation and Cochrane reviews

Reference	Language pair	Score (%)
Neveol et al. (2013) [11]	English → French Cochrane Reviews	BLEU: 40
Neves et al. (2016) [12]	English → Portuguese	BLEU: 33.37
	English → Spanish	BLEU: 31.11
Soares et al. (2018) [13]	English → Portuguese	BLEU: 48.51
	English → Spanish	BLEU: 37.93
Saunders et al. (2019) [14]	English → Spanish	BLEU: 48.93
Soares and Krallinger (2019) [15]	English → Portuguese	BLEU: 49.51
	English → Spanish	BLEU: 47.01
Peng et al. (2019) [16]	English → German	BLEU: 35.26
	English → French	BLEU: 38.29
	English → Chinese	BLEU: 37.09

For years 2018 and 2019, metrics refer to the WMT challenge of the respective years.

## Supplementary Materials

Supplementary Table 1. Machine translation and human back-translation of simple medication prescriptions
