# Supplementary material for: SciBabel: a system for crowd-sourced validation of automatic translations of scientific texts
Source: Genomics Inform. 2020 Jun 15;18(2):e21. doi: 10.5808/GI.2020.18.2.e21 (PMC7362948; doi:10.5808/GI.2020.18.2.e21)
Supplement: Supplementary Table 1. — Machine translation and human back-translation of simple medication prescriptions [file gi-2020-18-2-e21-suppl1.pdf]

Supplementary Table 1. Machine translation and human back-translation of simple medication prescriptions

Language	Machine translation	Human back-translation
Finnish	Ota kaksi tablettia suun kautta päivittäin, ellei heitä ole huimausta tai päänsärkyä.	Take two pills orally every day, **unless they are dizziness or headache.**
Korean	현기증이 나지 않는 한 매일 두 정을 입으로 가져갑니다.	As long as you don't feel dizzy **['lightheaded' missing]** move two pills to your mouth.
Portuguese	Tome dois comprimidos por via oral diariamente, a menos que esteja tonto ou tonto.	Take two tablets orally daily, unless you are dizzy or **dizzy.**
Italian	Prendi due compresse per via orale ogni giorno a meno che non abbia vertigini o stordimento.	Take two pills (by mouth) every day, unless you have vertigo or feel dizzy.
Spanish	Tome dos pastillas todos los días a menos que esté mareado o aturdido	Take two pills per day every day unless you feel dizzy or somewhat confused.
Japanese	めまいや立ちくらみがない限り、毎日2錠を服用してください	Please take two tablets every day unless you don't have dizziness or lightheaded
French	Prenez deux comprimés par jour, sauf si vous êtes étourdi ou étourdi	Take two pills every day unless you feel dizzy or **dizzy.**
German	Nehmen Sie jeden Tag zwei Tabletten ein, es sei denn, Ihnen ist schwindelig oder benommen	Take two pills every day by mouth unless you feel dizzy or lightheaded.
Russian	Принимайте две таблетки каждый день, если у вас нет головокружения или головокружения	Take two pills every day if you don't have vertigo or **vertigo.**
Chinese (Simplified)	除非头晕目眩或头晕目眩，否则每天服用两片	Take two tablets per day unless you feel dizzy or **dizzy.**
Ukrainian	Приймайте по дві таблетки щодня, якщо у вас не	Take two pills every day by mouth if you don't have dizziness

	запаморочилося чи не маєте голову	or lightheadedness.

Contraindications that have been incorrectly translated are highlighted in bold font.

The computer program made contraindication-related errors in six out of 11 translations
